# The Use of MRI and PET Imaging Studies for Prostate Cancer Management: Brief Update, Clinical Recommendations, and Technological Limitations

**DOI:** 10.3390/medsci7080085

**Published:** 2019-08-05

**Authors:** Margaret Mansbridge, Eric Chung, Handoo Rhee

**Affiliations:** 1 Department of Urology, Princess Alexandra Hospital, Ipswich Road, Woolloongabba, QLD 4102 Australia; 2 University of Queensland, St Lucia, QLD 4067, Australia; 3 AndroUrology Centre, Brisbane, QLD 4000, Australia

**Keywords:** prostate cancer, multi-parametric magnetic resonance imaging, prostate specific membrane antigen, positron emission tomography

## Abstract

Multi-parametric magnetic resonance imaging (mpMRI) and positron emission tomography (PET) using prostate-specific membrane antigen (PSMA) targeting ligands have been adopted as a new standard of imaging modality in the management of prostate cancer (PCa). Technological advances with hybrid and advanced computer-assisted technologies such as MR/PET, MR/US, multi-parametric US, and robotic biopsy systems, have resulted in improved diagnosis and staging of patients in various stages of PCa with changes in treatment that may be considered “personalized”. Whilst newer clinical trials incorporate these novel imaging modalities into study protocols and as long-term data matures, patients should be made aware of the potential benefits and harm related to these technologies. Published literature needs to report longer-term treatment efficacy, health economic outcomes, and adverse effects. False positives and negatives of these imaging modalities have the potential to cause harm and the limitations of these technologies should be appreciated. The role of a multi-disciplinary team (MDT) and a shared-decision-making model are important to ensure that all aspects of the novel imaging modalities are considered.

## 1. Introduction

Prostate cancer (PCa) is a ubiquitous disease, affecting a significant proportion of older males. Whilst the majority of patients with PCa will experience non-lethal localized disease, approximately 4% of males will ultimately succumb to the malignancy [1]. Commonly available investigative tools to diagnose and stage the disease include clinical history, examination, serum prostate-specific antigen (PSA), and transrectal ultrasound (TRUS) with biopsy of the prostate. Systemic staging is typically completed with abdominal and pelvic computed tomography (CT) or magnetic resonance imaging (MRI), and ^99^m-technetium labeled whole-body bone scintigraphy (BS). The gained information is then used to help stratify patients into different risk groups to determine the most suitable surveillance or treatment options from curative to palliative [2].

This strategy proved to be effective in selective patients such as in those with a low-risk localized disease with a low probability of progression and cancer-caused death [3]. On the other end of the spectrum, patients classified into high-burden metastatic disease using the above tools gained considerable survival benefit upon treatment with immediate chemotherapy and androgen deprivation therapy (ADT) [4,5]. Unfortunately, the conventional strategy does not appear to accurately diagnose and stage patients with localized intermediate- and high-risk disease or micro-metastatic disease. The deficiencies in staging have been exemplified in long-term treatment studies where patients who elected to undergo radical prostatectomy for localized disease developed subsequent metastatic disease in approximately 25% and biochemical recurrence (BCR) in 40% [6]. All of these patients were deemed to have had localized disease only using the conventional investigational repertoire.

Further, prospective PCa interventional studies such as the Scandinavian Prostate Cancer Group (SPCG-4) trial, Prostate Cancer Intervention versus Observation Trial (PIVOT), and Prostate Testing for Cancer and Treatment (ProtecT) trials have demonstrated that there are only a subset of patients such as those who are young and harbor high-risk disease or symptoms that would benefit most from the primary treatment whilst many others would be exposed to various dimensions of investigative and treatment adverse effects without significant survival advantage [6,7,8].

Overall, it has become apparent that the currently available tools for diagnosis and staging are unable to: 1) Detect metastatic disease early in the course of the disease process, and 2) selectively diagnose patients with PCa that is likely to cause harm.

There have been two recent major developments that have changed the PCa investigation and treatment paradigms even prior to the availability of long-term prospective data. Multi-parametric magnetic resonance imaging (mpMRI) and positron emission tomography (PET) using prostate-specific membrane antigen (PSMA) targeting ligands have been incorporated into various aspects of PCa investigations owing to its promising preliminary results. There are numerous other promising novel technologies that have either specific roles or are currently under investigation. For example, fluorodeoxyglucose (FDG) PET is used to stage rare neuroendocrine or small cell prostate tumours and to guide theranostic treatments in those with castrate-resistant prostate cancer (CRPC). Other PET imaging with radiopharmaceutical agents such as ^18^F-FACBC (Axumin^®^), ^18^F-choline, ^11^C-choline and, ^11^C-acetate have also proven to be useful for staging of patients, although at a higher PSA than with PSMA targeting ligands with a lower number of lesions of interest [9,10,11]. Moreover, there are hybrid and advanced computer-assisted technologies such as MR/PET, MR/US, multi-parametric US, and robotic biopsy systems that are very promising.

For the purpose of providing a salient and practical update around the most widely available novel technologies, only mpMRI and PSMA targeting PET/CT (commonly referred to as PSMA PET) will be discussed. This narrative review aims to discuss and evaluate the current evidence in the field of these two imaging modalities and make recommendations on its use at different stages of PCa.

## 2. Multi-Parametric Magnetic Resonance Imaging (mpMRI)

The quintessential diagnostic tool would selectively identify aggressive localized cancers early before they had the chance to develop locoregionally or develop metastatic disease. The test should be reproducible, undertaken in a standardized manner, cost-effective, and readily available. Although the most recent publications on mpMRI do not appear to demonstrate all the qualities of a perfect investigative tool, it does appear to have several advantages over the older imaging modalities.

Numerous studies have been published since the early 1980s regarding the potential role of MRI in screening and diagnosing patients with PCa [12]. Recently, improved MR technologies such as the development of 3-Tesla machines (therefore, the removal of use-limiting endo-rectal coils), faster image acquisition, and the addition of functional sequences, such as diffusion-weighted imaging (DWI) and dynamic contrast enhancement (DCE) have allowed for improvements in the quality and standardization of image acquisitions. The European Society of Urogenital Radiology (ESUR) helped develop a reporting framework called Prostate Imaging Reporting and Data System (PI-RADS). It commenced the mpMRI revolution in PCa and helped researchers and clinicians around the world use consistent MR sequence protocols and reporting [13]. Soon, numerous publications became available advocating for the routine use of mpMRI to improve the accuracy of clinically significant prostate cancer (csPCa) diagnosis, especially in those with negative TRUS biopsy and high suspicion of cancer [14]. Some authors suggested that mpMRI and MR targeted biopsy could omit systemic sampling to reduce the diagnosis of low-risk disease and thereby decrease the potential treatment harm to patients [15].

## 3. Positron Emission Tomography (PET) with Prostate-Specific Membrane Antigen (PSMA) Targeting Ligands

Prostate-specific membrane antigen is a type II integral transmembrane glycoprotein and is also known as glutamate carboxypeptidase II or *N*-acetyl-l-aspartyl-l-glutamate peptidase I [16]. The enzyme has been targeted with positron emission tomography integrated computed tomography (PET/CT) or hybrid MR (PET/MR) that detects gamma emission from radionucleotide with an affinity for the antigen. The glycoprotein can be upregulated by up to 1000-fold in malignant and metastatic PCa tissues with its expression linked to the aggressiveness and metastatic potential of PCa [17]. Owing to its promising preliminary results, PSMA PET has now widely become available. The technology commonly uses three different PSMA binding ligands: 1) ^68^Ga-PSMA-11 or ^68^-Gallium (Ga) Glu-urea-Lys-(Ahx) conjugated via the acrylic radiometal chelator ((*N,N*′-bis-[2-hydroxy-5-(carboxyethyl)-benzyl] ethylenediamine-*N,N*′-diacetic acid), 2) ^18^F-DCFPyL (2-(3-{1-carboxy-5-[(6-[(18)F]fluoro-pyridine-3-carbonyl)-amino]-pentyl}-ureido)-pentanedioic acid) or 3) ^18^F-DCFBC (*N*-[*N*-[(*S*)-1,3- dicarboxypropyl]carbamoyl]-4-[^18^F]fluorobenzyl-l-cysteine). Any of these agents can be used depending on the protocol of the service providers, although the majority of publications are based on ^68^Ga-PSMA-11. There are numerous other second-generation ligands with encouraging results currently under development and use in various clinical trials [9].

The molecular targeting strategy was initially marketed as ProstaScint^®^ in the 90 s using a radioisotope labeled antibody named ^111^Indium capromab pendetide [18,19]. Although promising, there was a significant delay in image acquisition after the administration of the radioligand. It has a long half-life and it targets the internal epitope of PSMA, thereby requiring internalization by PCa cells. In 2012, Afshar-Oromieh et al. first reported human imaging of localized PCa with ^68^Ga-PSMA-11 and PET/CT [20]. The Heidelberg group then reported a case series of patients with BCR [21] and found that it can identify metastatic lesions at a PSA level as low as 0.1 ng/mL with high contrast and accuracy. The routine use of this novel imaging modality in clinical practice was supported by relatively high maximum standardized uptake values (SUVmax) and tumor-to-background ratio, and simple radiopharmaceutical processing.

## 4. Prostate Cancer Diagnosis and Screening

There have been several large prospective clinical trials conducted to compare the diagnostic accuracy of mpMRI to detect csPCa. In the PROMIS study, a transperineal biopsy was used as the reference and performed in all patients with an elevated PSA in addition to TRUS biopsy of the prostate and mpMRI (Table 1) [22]. Among the 576 men suspected to have PCa, the sensitivity and negative predictive value (NPV) for csPCa diagnosis in the MR pathway was 93% (95% Confidence Interval (CI) 88–96%) and 89% (95% CI 83–94%) respectively. However, 11% of patients with no identifiable lesion on mpMRI were found to have significant cancer with a transperineal biopsy. On the other hand, the sensitivity and NPV were 48% and 74%, respectively, for the TRUS biopsy group. By using this technology as a screening tool, up to 27% of patients could have potentially avoided additional biopsy with an approximately 18% increase in the diagnosis of csPCa.

In a more recent PRECISION study, the authors prospectively compared the diagnostic accuracy of a standard TRUS biopsy to MR guided targeted biopsy only [23]. Of the 500 patients randomized, a larger number of patients with csPCa disease were identified with mpMRI and targeted biopsy compared to TRUS biopsy alone (38% vs. 26%). Whilst the risk of diagnosing non-csPCa was also lower (9% vs. 22%), the risk of radical treatment being offered was higher in the MR pathway (27% vs. 24%).

In contrast, the MRI-FIRST study found no significant difference in the detection of csPCa between systematic and targeted biopsies [24]. The study concluded that targeted biopsy was useful as an addition to systematic biopsy as it detected an additional 7.6% of csPCa. However, omitting systemic biopsy was discouraged as 20% of csPCa was detected by systematic biopsy only. As expected with systematic biopsy, the risk of a diagnosis of clinically non-significant cancer was significant (19.5% vs. 5.6%). The findings above have been supported by a recent systematic review of MRI pathways in the detection of localized PCa [25]. The sensitivity of the MRI pathway for diagnosis ranged from 67% to 100%, with the overall sensitivity at 78.3% (95%, CI 75–81.4%).

Rhee et al. first reported in a prospective study that PSMA PET also has a high detection rate in patients undergoing radical prostatectomy. All patients underwent PSMA PET and mpMRI prior to surgical removal and whole-mount correlation [26]. The study concluded that PSMA PET is equivalent to mpMRI in its accuracy in detecting localized PCa in this selective cohort. Both imaging technologies were found to be complementary such that the use of both yielded the highest positive predictive value (PPV) (95% vs. 80–90%). The findings have been supported by a recent systematic analysis where the pooled estimate of positivity in the prostate region was 90% for patients undergoing radical treatment [27]. 

**Limitations:** Whilst most studies have often demonstrated an increased diagnosis of csPCa via MR pathway, 10–25% of cancers were not identified by mpMRI. In addition, up to 80% of MR identified lesions did not harbor csPCa (depending on the grade of the MR lesion and the scale used). The cancer survival and health-economic outcomes are keenly awaited, although it would take one or two decades to demonstrate any improvement in cancer-specific mortality using this approach. The limitations with current literature include variations and inconsistencies in: 1) The image generation (e.g., MRI sequence protocols or the use of 1.5- or 3-Tesla magnets); 2) MRI reporting methods (e.g., Likert vs. PI-RADS v1 vs. PI-RADS v2); 3) the method of biopsies (e.g., cognitive vs. fusion vs. in-gantry); 4) the definition of clinically significant disease from biopsy (Gleason vs. size criteria); or 5) the definition of MR suspicious lesions (e.g., PI-RADS 3–5 vs. 4–5).

**Recommendations:** As the standardized image acquisition and reporting frameworks are evolving, certified radiologists with appropriate training and ability to liaise with fellow radiologists and patient-specific specialists are in the best position to enhance MRI reporting. There would be additional value in incorporating radiologists and nuclear medicine physicians in a multi-disciplinary team (MDT) setting. Patients with negative mpMRI require monitoring as a small but not insignificant proportion may harbor a clinically significant disease. The discussion with patients regarding PCa screening and investigations should include the potential benefits and harms of the new technologies. For example, the omission of biopsy based on mpMRI may result in a small risk of loss of opportunity to provide treatment in a timely manner. These imaging modalities clearly have a role in specific patient cohorts such as those who are at high risk or unable to undergo prostate biopsy (immunocompromised, coagulopathy, rectal pathology, abdominoperineal resection), or have had negative previous biopsies.

## 5. Treatment Planning/Primary Staging

In a recent systematic analysis of lymph node staging with PSMA PET and MRI in patients with intermediate to high-risk disease, the pooled sensitivity and specificity of PSMA PET were 0.65 (Confidence Interval (CI) 0.49–0.79) and 0.94 (CI 0.88–0.97) respectively [28]. The values were higher than with MRI at 0.41 (CI 0.26–0.57) and 0.92 (CI 0.86–0.95). Van Leeuwen et al. conducted a histological validation study to analyze 536 lymph nodes removed as a part of radical prostatectomy following the acquisition of PSMA PET images [29]. The mean diameter of PET-positive metastatic lymph nodes was 4.73+/−1.45 mm (range 3–9 mm) in comparison to 2.73 +/− 1.29 mm for PET-negative metastatic lymph nodes. The dimensions of metastatic nodes are substantially smaller than the 10 mm short-axis definition used by CT according to the Response Evaluation Criteria in Solid Tumours (RECIST) 1.1 [30]. The findings exemplified the size limitation of PSMA PET, which is partly explained by the spatial resolution of gamma cameras and the partial volume effect. Maurer et al. reported similar results where PSMA PET out-performed MR or CT with higher sensitivity, specificity, and accuracy (65.9%, 98.9%, and 88.5% vs. 43.9%, 85.4%, and 72.3% respectively) [31]. Despite the improved performance, only 27 of 41 patients with histologically proven lymph node metastatic disease were identified by the PET studies. 

In the setting of distant metastatic disease, PSMA PET and MRI could be used to help clarify indeterminant lesions on conventional imaging. The dilemma is created when there is a larger number of metastatic deposits than anticipated from conventional imaging. In a recent study by McCarthy et al., of 238 patients with BCR after radical prostatectomy PSMA PET identified lesions of interest in 74% whilst CT or BS identified lesions in only 16.4% [32]. Similar results were reported in a systematic review where PSMA PET regularly outperformed BS for osseous metastatic disease during primary staging [33]. The technology is currently being tested prospectively for primary staging in numerous studies including the proPSMA study (www.anzctr.org.au: ACTRN12617000005358). In this clinical trial, patients with high-risk PCa being considered for radical treatment are staged with conventional imaging as well as PSMA PET and followed up with for up to 54 months. 

Although mpMRI has been mostly studied for local staging and anatomical characterization, it does have a role in treatment planning. MRI has been found to be useful in further characterizing tumours anatomically. It provides information such as tumour size, location, and invasion of adjacent structures such as the capsule, neurovascular bundle, sphincter, pelvic floor, recto-prostatic angle, pelvic sidewalls, and seminal vesicles [34]. The information can be used to help tailor surgery, radiotherapy, or systemic therapy. However, in published retrospective reviews, mpMRI does not appear to help reduce the positive surgical margin status when used prior to surgery, perhaps due to the implementation of aggressive nerve-spare surgery in the absence of high-risk lesions on imaging [35].

**Limitations:** Limitations of PSMA PET include the non-standardized manufacture of ligands and its use, and the interpretation of data. Unlike mpMRI, there is currently no uniform interpretation guideline such as the PI-RADS. The Joint European Association of Nuclear Medicine/Society of Nuclear Medicine and Molecular Imaging published the first joint framework of recommendations of PSMA PET use in 2017 [36]. Through a proposed standardized system such as PSMA-RADS, inter-observer variability, and reproducibility may improve [37]. Also, previous large prospective studies such as CHAARTED and STAMPEDE did not define metastatic disease by MRI or molecular imaging [4,5]. As the studies concluded that patients with low-volume disease did not benefit from upfront chemotherapy based on conventional imaging, questions must be raised regarding the potential therapeutic benefit of chemotherapy in patients with high-burden metastatic disease defined by PSMA PET only.

One of the major pit-falls of PSMA based nuclear imaging is its physiological expression in lacrimal glands, salivary glands, liver, spleen, kidneys, small bowel, and nerves that may limit the interpretation near those anatomical sites. Pathologically, numerous benign and malignant tumours with revascularization may highly express PSMA [38]. A number of case reports have demonstrated the non-specific nature of PSMA PET, and its use must be in consideration of this [39].

**Recommendations:** An MDT consisting of a urologist, radiation oncologist, medical oncologist, radiologist, nuclear medicine physician, and allied health professionals is invaluable in challenging cases. For example, a patient with indeterminant lesions on systemic imaging may be characterized further with PSMA PET or MRI. The role of an MDT includes discussing the advantages and disadvantages of utilizing a novel imaging modality including its impact on delay in further management, potential stage-migration, and subsequent change in treatment aim. The implications of major treatment changes, such as treatment with intention-to-cure, or non-curative treatments, such as systemic therapy based on novel imaging, are unknown and the MDT discussion should reflect this concern. Further, patients with lesions identified on novel imaging only should be offered a biopsy if the clinical outcome is likely to change the management plan significantly.

## 6. Active Surveillance

Active surveillance (AS) is a safe and proven strategy for patients with low-risk PCa and longevity [40]. It allows for the selective treatment of patients who either demonstrate biological progression or those who are misclassified as low-risk. There are different protocols available around the world with subtle differences, however, all demonstrate excellent prostate cancer-specific mortality [3]. Multi-parametric MRI has been recommended in numerous AS protocols to rule out occult significant PCa that was unidentified during the initial work-up that may result in the loss of opportunity to cure early. Furthermore, there is emerging evidence to support its role in disease monitoring and replacing protocol-based biopsies.

The potential benefit of mpMRI in this cohort was initially assessed by Stamatakis et al. [41]. Twenty-nine percent (29%) of patients were subsequently upgraded or upstaged based on both mpMRI and targeted biopsy. In a more recent prospective study by Klotz et al., 273 patients enrolled in AS were randomized to either repeat TRUS or MR guided biopsy during follow-up [42]. MRI pathway showed a region of interest in 64% of patients. Subsequent upgrading was observed in 33% of patients compared with 27% in the standard TRUS biopsy group (*p* = 0.3). Of lesions on mpMRI, 26% were anterior lesions, which can be missed during TRUS biopsy. Overall, there was no difference between the two arms in the risk of upgrading within an AS protocol.

There are numerous other medium-sized studies with moderate follow up [43,44]. Gallagher et al. suggested that protocol-based biopsies could be replaced by repeat mpMRI, PSA, and DRE based on a follow up of 211 patients over 4.2 years [34]. The risk of upgrading on repeat biopsy in patients with negative mpMRI was 1.8%. In a recent meta-analysis of patients who underwent mpMRI within AS, the pooled sensitivity, specificity, NPV, and PPV were 60%, 76%, 75%, and 66% for upgrading [45]. The diagnostic values were higher for 3.0-Tesla machine utilizing studies. The potential benefit of repeat imaging with a median interval of 24 months was also tested by Eineluoto et al. [46]. In this selective cohort, 69% demonstrated mpMRI progression with either an increase in PI-RADS score, size, and/or the development of a new lesion. High-grade lesions have been correlated with disease upgrade in 47% of cases.

**Limitations:** The long-term safety, economic, or quality of life outcomes data are yet to be reported within AS protocols. Studies such as PRIAS MRI that are gathering information prospectively would be helpful. There is also no consensus on the acceptable level of mpMRI lesion grade for safe incorporation into the protocols.

**Recommendations:** The role of mpMRI within an AS protocol is evolving. Lesions of interest on mpMRI at the beginning and on follow up may be associated with csPCa that may require treatment. The selective use of mpMRI in those contraindicated to TRUS biopsies or in the young may be of benefit. Numerous professional bodies such as the European Association of Urology (EAU), the American Urological Association (AUA), and the National Institute for Health and Care Excellence (NICE) have advocated for the incorporation of mpMRI. The concept of omitting follow-up biopsy in place of mpMRI is still being tested, as is the safety of repeat imaging in place of biopsy. The small but real risk of omission of systemic, or any biopsy, using mpMRI, as well as potential increased need for further biopsy based on the MR findings should be discussed with patients.

## 7. Secondary Staging

Up to 40% of patients will develop BCR after primary treatment [47]. Whilst these patients have been staged to have a localized disease with conventional imaging, the ability to detect micro-metastatic disease is outside the scope of the imaging modalities. Once BCR has been established, salvage treatment in the form of surgery or radiotherapy may be offered to improve progression-free survival and overall survival. The long-term outlook is improved for selective patients with BCR after surgery when salvage radiotherapy is instigated before the PSA reaches 0.2–0.5 ng/mL [48,49].

The attention to PSMA PET was the most pronounced in this cohort as this is the only imaging modality that has a high detection rate of metastatic lesions even at a low PSA of <0.5 ng/mL [50,51]. In a recent systematic review, the authors compared the findings of all the imaging modalities reporting on the detection rate of metastatic disease in those with BCR. PSMA PET reported the highest localization of metastatic lesions with a PSA level <0.5 ng/mL (up to 65%), followed by ^11^C-choline PET/CT (up to 31.3%) [52]. Clinicians in practice have theorized that the ability to detect and treat micro-metastatic disease early would equate to a survival advantage. In a recent survey, 59% of patients considered for salvage treatment had a change of radiotherapy delivery dose and method based on molecular imaging [53]. With targeted salvage radiotherapy in addition to standard prostatic bed and pelvic radiotherapy, some case reports have reported durable PSA responses [54]. However, in the population who undergo salvage lymph node dissection based on PSMA PET, 5-year recurrence-free survival was only 6%, suggesting that there are likely to be smaller metastatic lesions in addition to the ones identified [55]. This is consistent with the studies discussed above regarding the size limitations of a detectable metastatic lesion by PET [33].

**Limitations:** Although PSMA PET is widely adopted, there is limited data on histological correlation from metastatic lesions due to its impracticality. Therefore, the true PPV (especially involving visceral and bony metastatic disease) is unknown for systemic staging. Longer-term prospective data will provide further clarification if PSMA directed management of cancer recurrence and oligo/poly-metastatic disease will yield survival advantage. There are currently over 100 clinical trials incorporating PSMA PET, 80 of which are actively recruiting with many of the trials addressing this issue (www.clinicaltrials.gov). In the setting of staging local recurrence or lymph node disease near the bladder, the accumulation of tracer within the bladder and subsequent halo-effect limits its use, although the second-generation PSMA radioligands are likely to improve this limitation [36]. For example, ^18^F-PSMA-1007 ligands have been shown to have minimal urinary excretion [56]. Furthermore, PSMA expression may be associated with a higher Gleason score and tumour stage since up to 10% of tumours do not over-express PSMA [57]. Therefore, a negative PSMA PET may not necessarily equate to non-metastatic disease.

**Recommendations:** In the BCR cohort, PSMA PET may identify metastatic foci early even when PSA is low. The detection rate increases with PSA and the findings may be used to aid clinical decision making, although any significant deviation from a standard treatment should be discussed in the MDT. For example, a patient who would have normally received salvage radiotherapy to prostatic bed and pelvic lymph nodes may be deemed incurable due to PSMA PET-only identified osseous lesions. Until robust long-term data becomes available, aggressive management of synchronous metastatic disease based on novel imaging should be considered experimental or performed within a clinical trial.

## 8. Conclusions

The novel imaging modalities, such as MRI and PET, have already been widely adopted into clinical practice. These technologies have shown much promise in various stages of PCa and are likely to continue to play important roles in the future (Table 2). The adoption of these technologies has resulted in improved diagnosis and staging of patients in various stages of PCa with changes in treatment that may be considered “personalized”. Whilst newer clinical trials incorporate these novel imaging modalities into study protocols and as long-term data matures, patients should be made aware of the potential benefits and harms related to these technologies. Published literature needs to report longer-term treatment efficacy, health economic outcomes, and adverse effects. False positives and negatives of these imaging modalities have the potential to cause harm and the limitations of these technologies should be appreciated. The role of an MDT and a shared-decision-making model are important to ensure that all aspects of the novel imaging modalities are considered.

## Figures and Tables

**Table 1:** Summary of recent MR-guided biopsy studies.

.	PROMIS (22)	PRECISION (23)	MRI-FIRST (24)
Number (n)	N = 576	N = 500 MR Pathway *n* = 252	N = 251
**Age** (years +/− standard deviation)	63.4+/−7.6	64.4+/−7.5	64 (59–68)
**PSA** (ng/mL +/− standard deviation or range)	7.2+/−2.9	6.75 (5.16–9.35)	6.5 (5.6–9.6)
**Clinical Stage** (TNM stage)	Less than or equal to cT2c	Less than or equal to cT2c	Less than or equal to cT2c
**Reference**	Transperineal biopsy and TRUS biopsy	MR targeted biopsy or TRUS biopsy	Systemic biopsy and MR targeted biopsy
**Definition of csPCa**	Gleason grade 4 or greater	Gleason grade 4 or greater	csPCa-A = Gleason grade 4 or greater *
**LESION Scale and Positive Predictive Values (%)**	Likert Radiology Reporting Scale		Likert Radiology Reporting Scale
	1 = 4	PI-RADS v2	
	2 = 12		
	3 = 21	1 = No biopsy	1 = No biopsy
	4 = 58	2 = No biopsy	2 = 6
	5 = 81	3 = 12	3 = 12
	(For Gleason score 4 + 3 or greater, or core length equal to or greater than 6mm)	4 = 60	4 = 31
		5 = 83	5 = 77
	
**Diagnostic Accuracy of mpMRI (%)**	418 of 576 positive with Likert Score 3–5	181 of 252 positive with PI-RADS 3–5	198 of 251 positive with Likert score 3–5
	Sensitivity = 88 (84–91)		
	Specificity = 45 (39–51)		
	PPV = 65 (60–69)	PPV = 53	
	NPV = 76 (69–82)		PPV = 41

* Other definitions of csPCa were tested. For comparison, a single definition of csPCa has been used for the table. Therefore, the figures may be different from the text. **Definitions**: N = number, NPV = negative predictive value, PI-RADS = Prostate Imaging Reporting and Data System, PPV = positive predictive value, TRUS = transrectal ultrasound, v = version.

**Table 2:** Summary of indications, contraindications, and limitations.

	mpMRI	PSMA PET
**Indications**	Active surveillance	
	Loco-regional and distant metastatic disease staging: Primary and Secondary	Loco-regional and distant metastatic disease staging: Primary and Secondary
	Clarification of indeterminant lesions on conventional imaging	Clarification of indeterminant lesions on conventional imaging
	Negative TRUS biopsy and clinical suspicious for undetected prostate cancer	Negative TRUS biopsy and/or mpMRI, and clinical suspicious for undetected prostate cancer
	Assessment of BCR near bladder neck	To determine suitability/treatment response for PSMA targeted theranostic treatment
	Treatment planning—respectability/risk of positive margin	
**Contra-Indications or Not Recommended for Interpretation**	Aneurysm clip	
	Embolization coil	
	Implanted cardiac device	
	Inner ear implant	
	Intracranial shunt	
	Metal prosthesis	
	Neuro/biostimulator	
	Ocular metallic foreign body	
	Claustrophobia	
	Contrast allergy	Contrast allergy
	Unable to lie down	Unable to lie down
**Use in Special Circumstances**	Contraindicated to prostate biopsy (Coagulopathy, rectal pathology, anterior-perineal resection, immunocompromisation)	Contraindicated to prostate biopsy (Coagulopathy, rectal pathology, anterior-perineal resection, immunocompromisation) Renal impairment
**Limitations to Interpretation of Images**	Haemorrhage Prostatitis Fibrosis Atrophy Radiotherapy Hormonal therapy Renal impairment Prosthesis	Local recurrence near bladder neck or a metastatic deposit near bladder. Non-PSMA producing tumour including aggressive tumours such as small cell, neuroendocrine and ductal carcinoma types. Physiological expression in lacrimal or salivary glands, liver, spleen, kidneys, small bowel and ganglia. Benign and malignant tumours with neovascularisation.
